# Caring for people with heart failure and many other medical problems through and beyond the COVID‐19 pandemic: the advantages of universal access to home telemonitoring

**DOI:** 10.1002/ejhf.1864

**Published:** 2020-06-01

**Authors:** John G.F. Cleland, Robyn A. Clark, Pierpaolo Pellicori, Sally C. Inglis

**Affiliations:** ^1^ Robertson Centre for Biostatistics & Glasgow Clinical Trials Unit, Institute of Health and Wellbeing University of Glasgow, Glasgow Royal Infirmary Glasgow UK; ^2^ South Australian Health and Medical Research Institute (SAHMRI) Flinders University Adelaide Australia; ^3^ Faculty of Health University of Technology Sydney Sydney Australia

**This article refers to ‘Telemonitoring versus standard care in heart failure: a randomised multicentre trial’ by M. Galinier *et al*., published in this issue on pages 985–994.**

*Never let a ‘good’ crisis go to waste!*

*(attributed by some to Winston Churchill on planning*

*to set up the United Nations during World War II)*

In this issue of the Journal, Galinier *et al*.^1^ report a randomised trial of care supported by home telemonitoring including almost 1000 patients with heart failure. Telemonitoring consisted of questions about symptoms and daily weights. Data were relayed to a secure server, which generated alerts, to which nurses responded, during routine working hours, by advising patients whether they should contact their family practitioner or cardiologist. Compliance with measuring weight was often poor. We are not told how many patients contacted a doctor, what advice they received, or whether they complied with it. This complex chain of communication is only as strong as its weakest link.

The trial was neutral for its composite primary endpoint, unplanned hospitalisation for heart failure or all‐cause mortality (rate ratio 0.97; *P* = 0.80), and for all pre‐specified secondary endpoints. A further analysis, focussing on first unplanned hospitalisation for heart failure, suggested a modest improvement (hazard ratio 0.79; *P* = 0.044), that was driven by larger effects in those who weighed themselves regularly or had greater functional limitation or who were more socially isolated. Some will view this trial as further evidence that home telemonitoring is ineffective for heart failure, ignoring the overall positive effect identified by systematic reviews.^2^, ^3^, ^4^ Others will suggest the trial was neutral because of inadequate technology, lack of a robust and timely response to alerts, insufficient patient motivation and the problems inherent in conducting trials of service re‐design. Ultimately, a system that is cost‐efficient, user‐friendly and person‐centred does not need to show that it improves outcome; it only needs to show that it is not inferior to traditional ways of delivering care.^5^ Now, more than ever, this evidence is reassuring.

The COVID‐19 pandemic is now revolutionising attitudes to remote patient follow‐up; widespread scepticism has switched to near‐universal enthusiasm and rapid adoption into routine care.^6^ The drive comes from both health professionals and patients, who want to comply with social distancing whilst ensuring continued delivery of good healthcare. This applies especially to patients with heart failure. In retrospect, it is a great shame that home telemonitoring was not already routine before the pandemic struck. This would have saved billions of healthcare ‘dollars’ worldwide as well as the stress of rapidly implementing telehealth without established infrastructure or protocols. Preparing for, rather than reacting to, a crisis seems wise, but is it rational to configure healthcare for non‐communicable diseases in future decades around the risk of pandemic infections? Perhaps not, unless there are other advantages to telecare.

All those involved in telemonitoring must be motivated to support the service, most importantly the patient. Patient attitudes have changed hugely with the advent of the COVID‐19 pandemic; many are refusing to come to a healthcare facility, in primary or secondary care, even if they are unwell. Many are now eager to have healthcare delivered at home. Although some may not cope with the technology, most will have a family member or carer who can. Ultimately, home telemonitoring will not work for every patient but if it works for some, then resources can be targeted more effectively for all.

The technology must be easy to install, intuitive to users and provide robust communication. Achieving uniformity across telehealth platforms for healthcare providers and medical specialities would be a bonus for patients; using a different system to manage each of a patient's medical problems is confusing, costly and impractical. Smartphones are widely available in high‐, middle‐ and most low‐income countries and solve many of these problems. Multi‐user systems in care homes or community tele‐kiosks can make even more efficient use of equipment when social distancing is not possible or not required. Voice interactive systems annoy many people and should be avoided until they can deal with local dialects. Devices should connect to systems wirelessly. Systems should provide a flexible set of modules that can be aligned to the patient's medical needs, commonly including hypertension, diabetes, coronary artery disease, atrial fibrillation, lung disease, renal dysfunction, heart failure, and mental health. Perhaps everyone with a chronic disease or everyone of retirement age should be offered telemonitoring. Ultimately, telehealth could become another standard household utility, just like electricity, gas, or the internet, providing education and trusted advice for people of all ages. Where healthcare is provided by the State or an insurance company, provision of the internet and telehealth should attract no additional cost to the patient. Better to have immediate triage by telehealth to the need for and most appropriate healthcare provider, whether that is a pharmacist, physiotherapist, family practitioner or specialist, rather than waiting days for an appointment before sitting in a waiting room for hours for a 5‐min consultation with a doctor, only to be referred on to someone else. What would you prefer?

Most routine healthcare can be automated. For problems such hypertension and atrial fibrillation, patients can do non‐invasive checks, which can be fed into their electronic health record, where guideline‐driven algorithms can advise the patient and local pharmacy about which tests and treatments are required. For patients who already have a device implant, such as a loop recorder, pacemaker, defibrillator or pulmonary artery pressure monitor, even more information can be obtained. A doctor needs only enter the healthcare loop when required. Doctors and nurses should facilitate such automation rather than be sceptical and a barrier to efficient care. Physical contact may be more important for the psychology of health professionals than for patients, who may just want good care from someone they trust and can access without needing to wait weeks or months for an appointment. Clearly, some people will prefer more traditional forms of care and, for the complex cases that algorithms cannot currently manage, consultations may take longer. Rapid adoption of telehealth during the pandemic has created an opportunity to re‐design how care is delivered and by whom (mainly patients) that should be grasped.

Many trials of telemonitoring for heart failure have attempted to predict and manage episodes of decompensation; this ‘crisis management’ strategy has produced inconclusive results.^7^ The high rate of false‐positive alerts is its Achilles' heel. Rather than trying to detect something going wrong and fixing it, a ‘health maintenance’ strategy declares an ideal target for an individual and adjusts treatment to maintain them as close to ideal as possible. This strategy avoids the problem of false alerts and involves the patient more closely in their care. Better control of congestion will have favourable effects of atrial and ventricular remodelling, arrhythmias and pulmonary hypertension, which should improve prognosis.^8^, ^9^, ^10^ A health maintenance strategy can be personalised and delivered by a friendly, local healthcare team. Data from hundreds of patients can be managed by automated systems, requiring staff input for only a few hours per week and can readily be integrated with physical visits in the community or hospital. Remote monitoring does not have to mean remote care. A crisis management strategy requires a 24 h, 7‐day per week service. This might be manageable at a regional or national level but too expensive for local services, unless delivered as an extension of existing facilities such as coronary care units. The two approaches are not mutually exclusive, but the former is likely to be the more feasible and cost‐effective component.^11^ Patients often get bored with telemonitoring systems that provide no feedback and do not seem to ‘do’ anything. A health maintenance strategy provides much greater patient engagement and motivation through both education and patient action.^12^, ^13^ Educating patients, empathetically, about what treatment targets should be achieved, whether that is weight, heart rate or blood pressure, empowers them to participate in the development and delivery of care. Patients who are unaware of their care plan may struggle to help doctors deliver it. Telemonitoring can deliver health education in many formats; infographics, videos, gaming, quizzes, and inform patients of what tests they need and why and when. Measurements can also be used to determine daily doses of diuretics and some other medications.^14^ The habit of giving the same diuretic dose every day arises from the lack of resources and technology to monitor requirements.

Telemonitoring should be time efficient. Dentists recommend that people should spend at least 2 min twice a day brushing their teeth. A cardiovascular health check takes less time. Daily checks are probably not required for stable patients; text reminders can be scheduled less often. The focus should be on acquiring therapeutically actionable data, such as symptoms, heart rate and rhythm, weight, or more sophisticated measurements of congestion if available (*Table* *1*). Pulse oximetry might be useful for managing respiratory disease including COVID‐19 infections of intermediate severity. Haematocrit, serum potassium and creatinine are actionable but no reliable, affordable method of home measurement currently exists, but technology is being developed. Local phlebotomy services, with home visits if necessary, would be a simple and inexpensive alternative. Video‐consultation can often replace clinic visits. Indeed, armed with a wealth of patient‐recorded information, linked to electronic healthcare records and supported by machine‐learning algorithms, the consultation, for both patient and health professional, may be more rewarding than a physical visit. However, video‐consultation is time‐consuming and probably not the most effective or efficient way that telemonitoring can support patient self‐management.

**Table 1:** Actionable measurements and interventions required for care of patients with heart failure

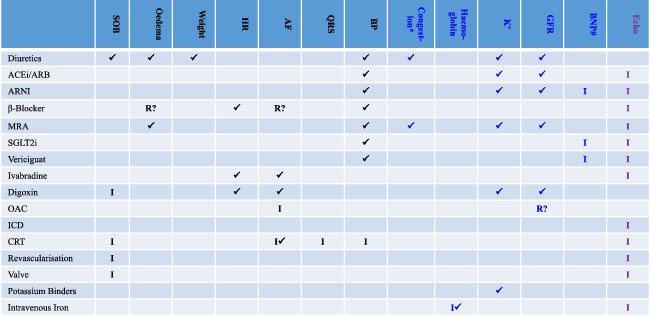

ACEi, angiotensin‐converting enzyme inhibitor; AF, atrial fibrillation; ARB, angiotensin receptor blocker; ARNI, angiotensin receptor–neprilysin inhibitor; BNP, B‐type natriuretic peptide; BP, blood pressure; CRT, cardiac resynchronisation therapy; Echo, echocardiography; GFR, glomerular filtration rate; HR, heart rate; ICD, implantable cardioverter‐defibrillator; K^+^, serum potassium; MRA, mineralocorticoid receptor antagonist; OAC, oral anti‐coagulant; QRS, QRS width on the electrocardiogram; Revascularisation, coronary revascularisation; SGLT2i, sodium–glucose co‐transporter 2 inhibitor; SOB, shortness of breath; Valve, valve procedure– mitral or aortic.

✓= required for monitoring during follow‐up; I = mainly for deciding on initiation or investigation; R? = consider reducing or temporarily stopping.

Black = currently widely available technologies (e.g.: ECG/sphygmomanometer). Blue = technology with limited availability for use at home (e.g. bio‐impedance, blood tests). Echo is in purple, because although home monitoring is unlikely in the near future, it plays an essential role in identifying reduced left ventricular ejection fraction and valve disease and therefore in treatment selection.

^#^BNP is a useful marker of poorly controlled congestion/heart failure and the need for further investigation/treatment.

*Water retention leading to oedema (an increase in water in the tissues). Detection and treatment of subclinical oedema can prevent symptoms and improve prognosis.

There are other important consequences of switching from a traditional model of care. Home telemonitoring enables patients to be part of the workforce, delivering truly individualised healthcare.^15^ Even in countries with ostensibly free healthcare, the costs of getting to clinics are usually borne by the patient. Travel to and waiting at a clinic can easily take up most of a patient's day. Other hidden costs include air pollution and traffic congestion and the costs of building clinic space and carparks. Also, telemonitoring requires an associated electronic health record that enables machine learning that can generate advice for both patients and health professionals. Clearly, some people will feel uncomfortable about their data being used like this, but their concerns should not deny benefit to others. Informed patients should choose who has access to their data and for what purpose. This could benefit billions of people worldwide. Regulations that pay more attention to the worried‐well than to patients who are in need must be avoided. The pandemic has shown us the public's view on this issue.

In summary, adoption of home telemonitoring as a routine clinical service for most medical problems will improve the efficiency and quality of care, reduce demands on patients, and reduce healthcare costs and environmental pollution. It will also reduce the spread of infections, whether that is the misery of seasonal rhinovirus, annual influenza, or a lethal pandemic. Let's do it—now!

## Funding

SCI is supported by a Future Leader Fellowship, Heart Foundation of Australia.

**Conflict of interest:** J.G.F.C. has received honoraria from Abbott, Medtronic and Philips for advisory boards. All other authors report no conflicts of interest.
